# Early outcomes of the “Chimney” commando procedure in the small aortic and mitral annuli

**DOI:** 10.3389/fcvm.2023.1139771

**Published:** 2023-07-24

**Authors:** Mingyuan Yang, Wenhao Liu, Laichun Song, Jingcheng Wu, Yong Xiao, Yuhang Liu, Liang Tao

**Affiliations:** Department of Cardiac Surgery, Asia Heart Hospital of Wuhan University, Wuhan, China

**Keywords:** commando procedure, small annulus, chimney technology, double valve replacement, prosthesis-patient mismatch

## Abstract

**Background:**

Commando procedure, the surgical replacement of the mitral and aortic valves combined with reconstruction of the fibrosa fibrous body, is a technical challenge in patients with small aortic and mitral annuli. In this study, we evaluated the safety and early outcomes of the “Chimney” modality of the Commando procedure, in patients with small aortic and mitral annuli, after prior valve surgery, using a self-assembled valved conduit.

**Methods:**

From April 2021 to April 2022, 30 consecutive cases of the “Chimney” Commando procedure, with a self-assembled valved conduit and other combined cardiac procedures, were fully performed for re-operative patients with small aortic roots. Data were obtained through a medical record review, at the Asian Heart Hospital in Wuhan, China.

**Results:**

The patient's mean age was 52.7 ± 13.53 years, with 93.3% females. All patients had a previous heart valve surgery, 90% of which had double valve replacement (DVR). Hospital death occurred in 3.3% (*n* = 1) of the patients, due to malignant arrhythmias and multiorgan failure. Postoperative echocardiogram exams showed that the sizes of the aortic and mitral valve prostheses were 24.23 ± 1.60 mm and 28.33 ± 1.21 mm, respectively. All patients had intact intervalvular fibrosa (IVF) repair and no patient had any aberration in the left heart chamber communication. With the exception of one postoperative sick sinus syndrome and one re-sternotomy for bleeding, there were no significant postoperative complications, such as mortality, renal failure requiring ongoing dialysis, or mediastinitis. Echocardiography exams in the sixth postoperative month showed that the mean gradients of the aortic and mitral valves were 16.26 ± 6.44 mmHg and 11.24 ± 4.90 mmHg, respectively.

**Conclusions:**

In comparison with the standard Commando operation, the early outcomes and safety of the “Chimney” Commando procedure proved to be a feasible therapeutic option for patients with small aortic and mitral annuli, after prior valve operations. This approach enables the enlargement of the aortic and mitral annuli and the implantation of the necessary valve prosthesis.

## Introduction

Double valve replacement (DVR), the replacement of both the aortic and mitral valves, has been safely and smoothly performed in patients with valvular disease. However, prosthesis–patient mismatch (PPM) is a risk in patients with small-valve annuli (including the aortic and mitral annuli) (1). Especially in patients with small aortic roots, PPM is associated with decreased hemodynamic function, decreased regression of left ventricular masses, increased number of cardiac events, and poor survival rates. The 21 mm or smaller size prostheses were the most frequently implanted in patients with a small aortic annulus. According to the aortic valvular size, the mitral valve sizes were 25 mm or smaller, which may provide acceptable hemodynamic performances and midterm clinical outcomes (2). Nevertheless, those patients had an elevated tendency for anterograde mean and peak gradient (PG) values. Reoperation, which can include the redo-aortic valve replacement (AVR) or the redo-mitral valve replacement (MVR), should be carried out when required. David et al. (3) reported the conjunction of AVR, MVR, and the reconstruction of the intervalvular fibrosa (IVF), which is known as the Commando procedure. When patients suffer from unusual conditions, such as severe aortic and mitral annular calcification involving the IVF, the Commando procedure is performed (4). This surgical technique consists of a double valve replacement in patients with small aortic and mitral annuli (or double valve reoperations in which no IVF is available), followed by the excision of both valves. In our center, 30 patients with small aortic and mitral annuli successfully performed the “Chimney” Commando procedure after prior valve operations, with excellent clinical results.

## Patients and methods

Data were retrospectively analyzed from Asian Heart Hospital Wuhan and this study was approved by the institutional review board of Wuhan Asia Heart Hospital and was in compliance with the Health Insurance Portability and Accountability Act regulations and the Declaration of Helsinki. The institutional review board waived the need for individual patient consent. All patients who underwent the “Chimney” Commando procedure were included.

**Figure 1:**
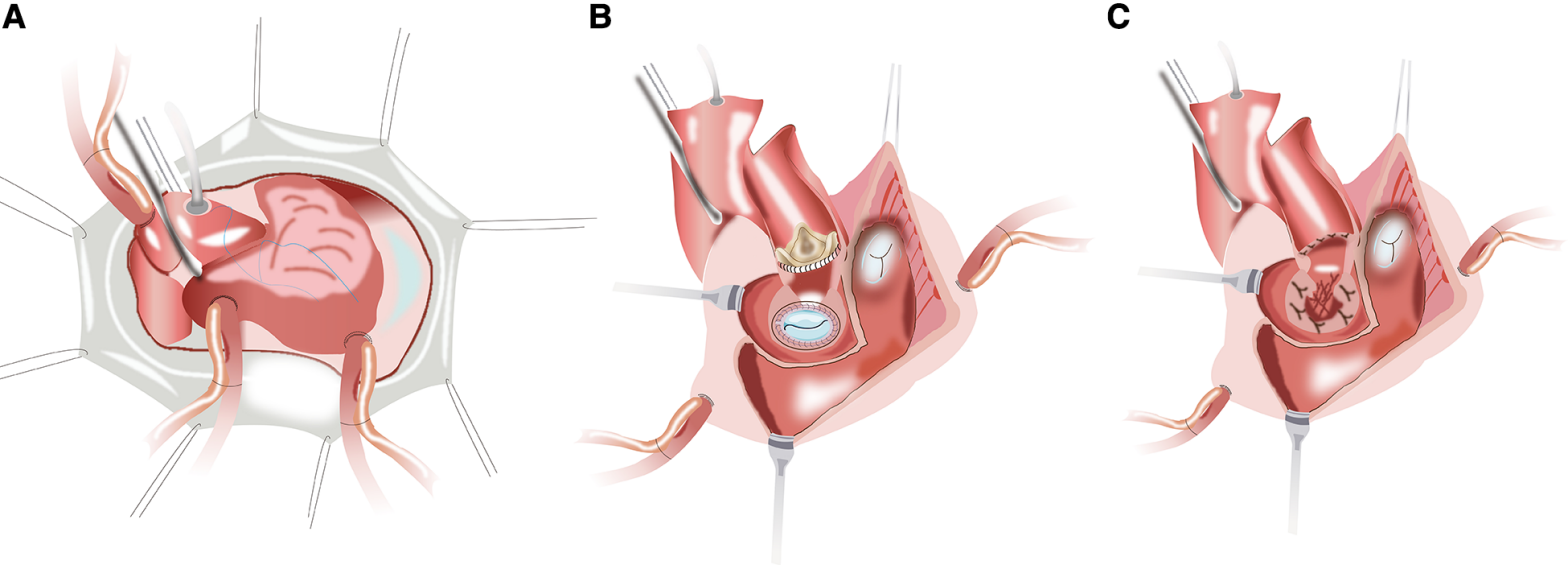
(**A**) the planned hockey-stick aortotomy and the extended superior transseptal incisions. (**B,C**) The aortotomy extends to across the left/nocoronary commissure into the sewing cuff of the mitral valve prosthesis, and the vertical incision in the fossa ovalis extends across the superior interatrial septum to open both the inflow and outflow of the left ventricle.

After a routine re-sternotomy, the patient is administered systemically Heparin (300–400 Units/Kg). Cannulation is performed through the distal ascending aorta and both superior and inferior venae cavae. The CPB is partially initiated when the activated clotting time (ACT) exceeds 450 s. A cardioplegia perfusion needle was inserted into the root of the aorta. The aortic root was demobilized from the right ventricular outflow tract and left atrial. After aortic cross-clamping, myocardial protection was provided with antegrade and retrograde cold HTK cardioplegia. Towards the commissure between the non- and left coronary sinuses of Valsalva, an oblique aortotomy is performed. The incision in the left atrial wall at the aortic root was then extended cranially to increase surgical accessibility (Figure 1A). The prior aortic prosthesis was removed, and the annulus underwent significant debridement, resulting in a complete or at least partial loss of annular tissue. Following the snaring of the superior and inferior venae cavae, an oblique right atriotomy is performed from the right atrial base to the inferior vena cava cannula, avoiding the area of the sinus node. A vertical incision in the fossa ovalis was created, extended caudally towards the inferior venae cavae and cephalad towards the aortic root to join the first incision, which caused the left ventricle's inflow and outflow pathways to combine into one large orifice (Figure 1B). The previous mitral prosthesis is excised and the annulus is debrided thoroughly (Figure 1C).

**Figure 2:**
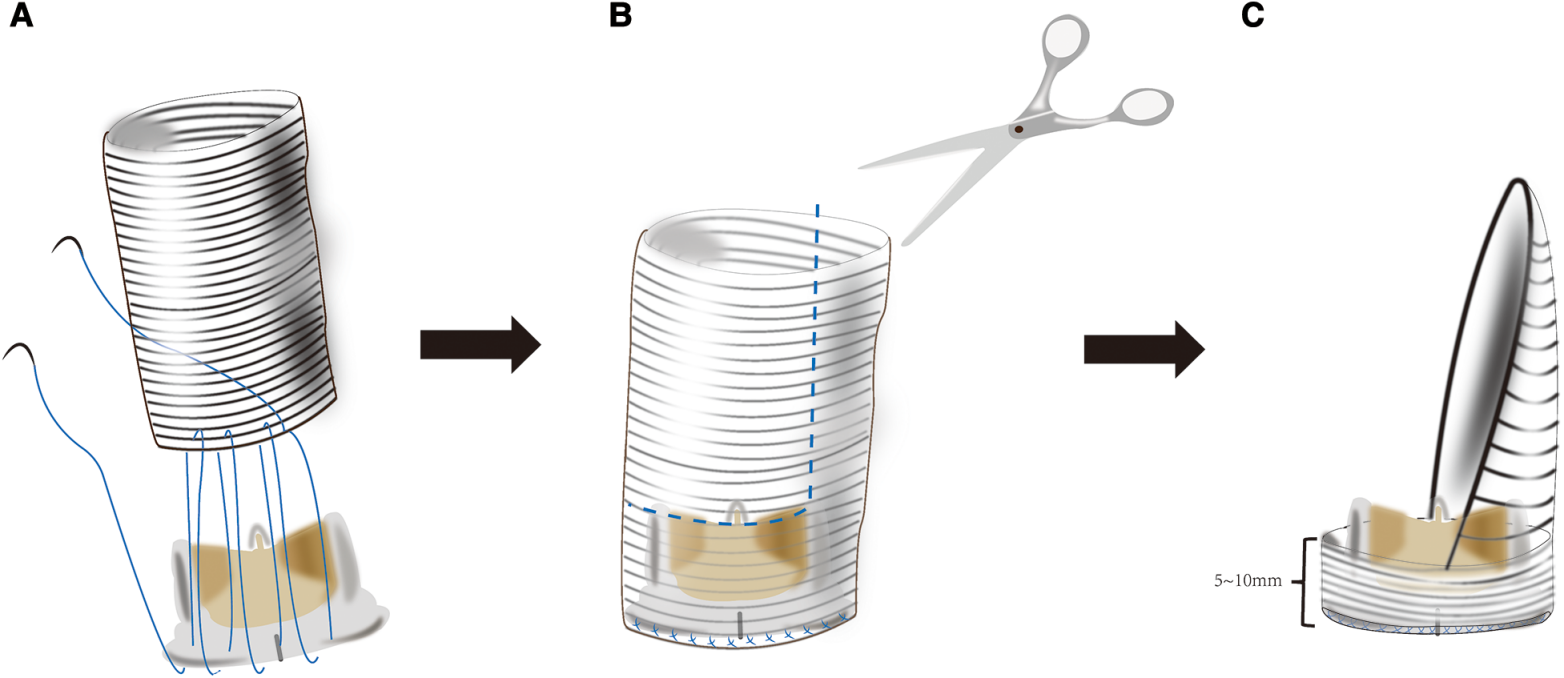
(**A**) the mitral valve prosthesis is sutured inside of a tubular woven polyester vascular graft 3–4 cm long and adequate diameter to accommodate a desired valve 5–10 mm from one of its ends. (**B,C**) The tubular woven polyester vascular graft is trimmed to a valved conduit with a 5–10 mm skirt end above the valve and a woven polyester vascular patch.\.

The next critical step is to assemble the new mitral valvular conduit. A woven polyester vascular graft (InterGard; Maquet Cardiovascular, La Ciotat, France) and any valve prosthesis including the bileaﬂet St Jude (St Jude Medical, Inc, St Paul, MN) prosthetic valve and Medtronic Aortic AP (Medtronic Inc, Minneapolis, MN) could be used to assemble the new valvular conduit (5). The valve is fixed within the vascular graft with running 4-0 polypropylene sutures. About three-fourths of the circumference of the self-assembled valvular conduit leaves an approximately 5–10 mm free margin above the valve. According to the need for surgery, the margin above the valve's length is chosen. The remainder of the self-assembled valvular conduit leaves adequate length to crop a vascular patch cutting into the sutured aorta (Figure 2).

**Figure 3:**
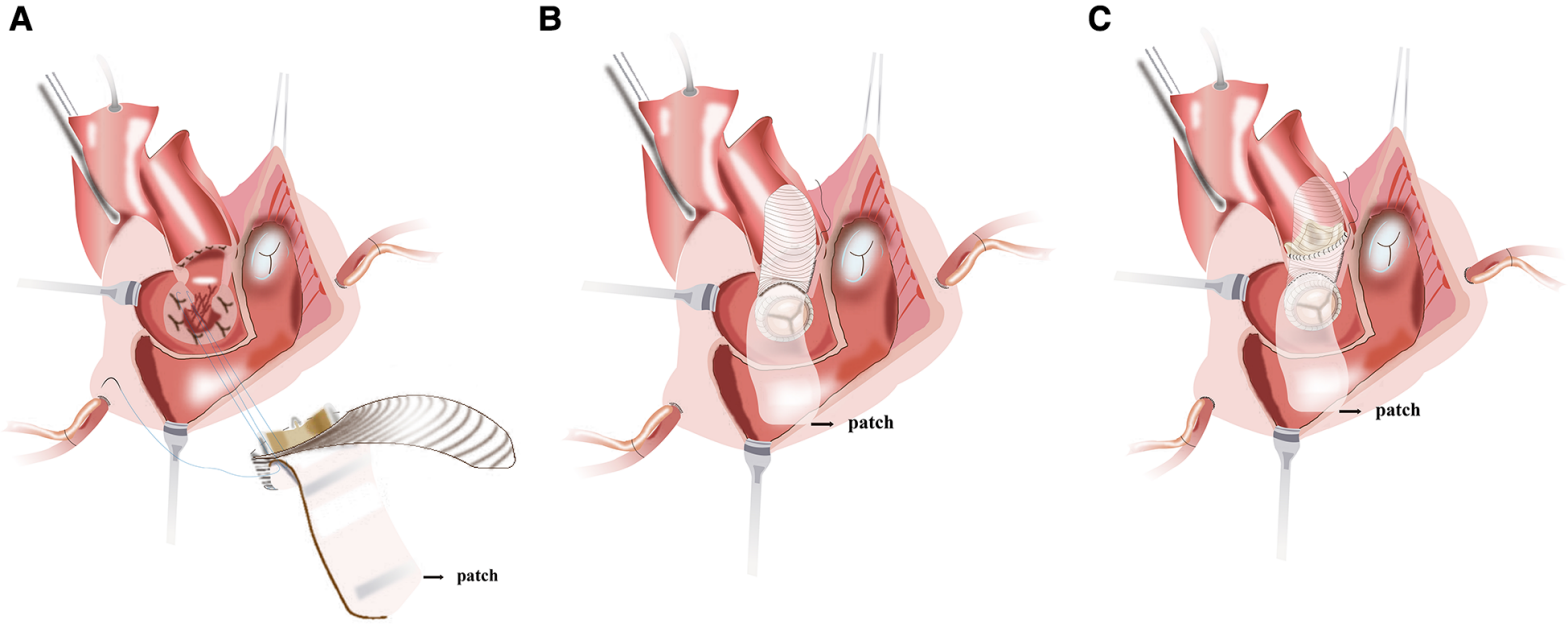
(**A**) the valved conduit is secured along the posterior mitral annulus or the endocardium of the posterior wall of the left atrium. (**B**) The vascular patch of the valved conduit fills the gaps in the aortic annulus and in the proximal ascending aorta and enlarges the aortic root. (**C**) The aortic prosthesis is placed and secured to the annulus and the vascular patch.

**Figure 4:**
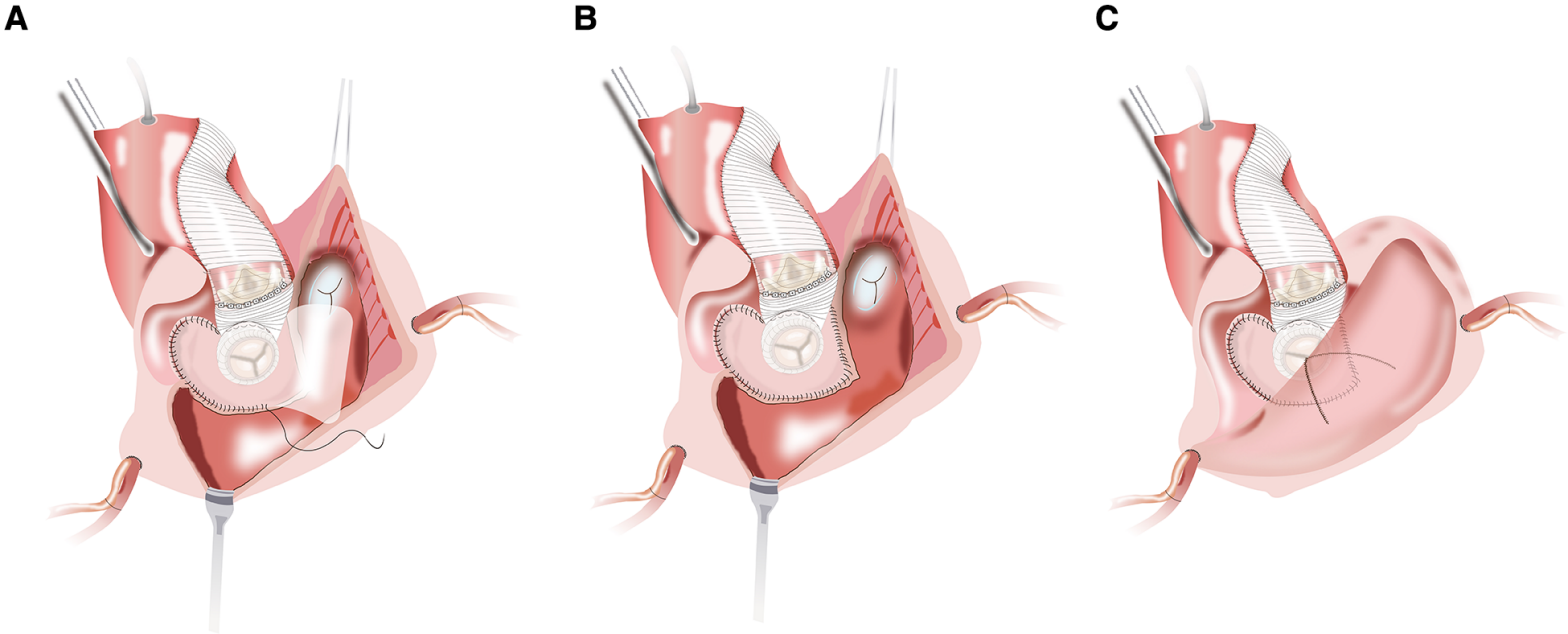
(**A**) the aortotomy is closed with the vascular patch. (**B**) The reconstruction mitral annulus's remainder of the patch is used to close the left atrial dome and interatrial septum. (**C**) Right atrial closure and complete restoration of the aortic and mitral annuli.

To construct the intervalvular fibrosa with patch and fix the self-assembled valvular conduit is performed. An appropriate-sized patch for IVF reconstruction and later closure of the left atrial dome is selected. Due to its accessibility and ease of handling, we prefer using bovine pericardium as a patch material. During the procedure of MVR, about three-fourths of the circumference of the self-assembled valvular conduit is secured to the native mitral annulus with a continuous stitch of 2/0 Prolene. The remainder of the self-assembled valvular conduit is stitched to a reconstructed annulus with bovine pericardium (Figure 3A). Unlike the Manouguian technique (6), the trimmed vascular patch of the self-assembled valvular conduit fills the gaps in the aortic annulus and in the proximal ascending aorta to make the vascular patch part of the aortic valve annulus (Figure 3B). The appropriate aortic prosthesis is chosen, and AVR was performed. The usual approach then involves fastening the aortic prosthesis along most of the aortic annulus. Sutures are inserted into the prosthesis sewing ring in the vicinity of the vascular patch from the outside of the patch (Figure 3C). The trimmed vascular patch is then used to close the aortotomy incision performed (Figure 4A). The reconstruction mitral ring's remainder of the patch is then tailored and used to close the left atrial dome (Figure 4B). When the heart is still silent, the area of the right atrium that is tightly connected to the left atrial dome is closed. After the cross-clamp is removed, the right atriotomy is closed in the standard procedure (Figure 4C). Temporary epicardial pacing wires are routinely placed. The incision is typically closed in layers after chest tubes are inserted and hemostasis is obtained. After transfer to the ICU, anticoagulation is routinely administered as soon as possible to avoid thrombosis (Supplementary Video 1).

Data analysis was performed with R software (version 4.2.1; http://www.Rproject.org). All categorical data were expressed as proportions and continuous variables were expressed as mean $\bar{\chi }\;\pm \;\mathrm{SD}$ or median with date range. The reported statistical significance levels were all two-sided, with statistical significance set at. 05.

## Results

### Cohort characteristics at the time of surgery

All patients had prior heart valve surgery. One patient (3.3%) was admitted for a third surgery and another patient (3.3%) was admitted for a double valve repair. As a consequence of heart failure caused by valve prosthesis stenosis, 22 patients needed a second surgery. Out of the 24 patients (80.0%) who had undergone any surgery within the previous five years, two needed a second surgery for an aortic root abscess. In the first month after the most recent valve surgery, the mean gradients of the aortic and mitral valves were 20.86 ± 2.53 mmHg and 13.17 ± 2.21 mmHg, respectively. Baseline transthoracic echocardiography exams showed aortic valve and mitral valve peak gradients of 82.57 ± 24.48 mmHg and 19.43 ± 8.97 mmHg, respectively. The mean diameter of the aortic valve annulus was 21.03 ± 1.90 mm. PPM was found in both valve prostheses. The effective orifice area index (EOAI) values for the aortic and mitral valves were 0.69 ± 0.06 cm^2^/m^2^ and 0.86 ± 0.05 cm^2^/m^2^, respectively. None of the first surgical replacement aortic valve prosthesis sizes was larger than 21#. Mitral valve prosthesis sizes were 25# (78.6%) and 27# (21.4%) (Tables 1, 2).

**Table 1:** Preoperative characteristics.

Variables	*n* (%) or mean ± SD
Demographics	
Age (years)	52.7 ± 13.53
Female	28 (93.3)
Body surface area (m^2^)	1.62 ± 0.15
NYHA III or IV	16 (53.3)
Non-cardiac comorbidities	
Hypertension	5 (16.7)
Diabetes	1 (3.3)
COPD	1 (3.3)
CKD	2 (6.7)
Stroke	3 (10.0)
Cardiac comorbidities	
Prior myocardial infarction	1 (3.3)
CAD	2 (6.7)
Atrial ﬁbrillation	11 (36.7)
Infective endocarditis	2 (6.7)
Prior cardiac surgery	
First reoperation	*n* = 29 (96.7)
Double valve replacement	27 (90)
Double valve repair	1 (3.3)
Aortic valve replacement	1 (3.3)
Second reoperation	*n* = 1 (3.3)
Double valve replacement	1 (3.3)
Postoperative month of the last valve surgery	
Aortic valve MG (mmHg)	20.86 ± 2.53
Mitral valve MG (mmHg)	13.17 ± 2.21
The last valve surgery period elapsed	
<5 years	24 (80.0)
5–10 years	3 (10.0)
>10 years	3 (10.0)
Aortic valve prosthesis	*n* = 29
St. Jude-17#	2 (6.9)
St. Jude-19#	10 (34.5)
St. Jude-21#	12 (41.4)
SJMR-19#	4 (13.8)
Med. Han-21#	1 (3.4)
Mitral valve prosthesis	*n* = 29
St. Jude-25#	23 (79.3)
St. Jude-27#	6 (20.7)

COPD, chronic obstructive pulmonary disease; CKD, chronic kidney disease; CAD, coronary artery disease; St. Jude, St Jude Medical; SJMR, St. Jude Medical Regent; Med. Han, Med. Hancock.

**Table 2:** Preoperative echocardiogram.

Variables	*n* (%) or mean ± SD
Echocardiographic data	
Aortic valve MG (mm Hg)	43.24 ± 17.93
Aortic valve PG (mm Hg)	82.57 ± 24.48
Aortic valve annulus diameter (mm)	21.03 ± 1.90
Mitral valve MG (mmHg)	19.43 ± 8.97
Mitral valve PG (mmHg)	21.73 ± 5.69
EOAI (cm^2^/m^2^)	
Aortic valve	0.69 ± 0.06
Mitral valve	0.86 ± 0.05
LAD (cm)	4.97 ± 1.05
LVDd (cm)	4.75 ± 0.37
IVSd (cm)	1.49 ± 0.17
LVPWd (cm)	1.36 ± 0.12
Ejection fraction (%)	53.45 ± 5.19

MG, mean gradients; PG, peak gradients; EAOI, effective orifice area index; LAD, Left atrial diameter; LVDd, left ventricular end diastolic diameter; IVSd, interventricular septal-end diastolic; LVPWd, left ventricular posterior wall-end diastolic.

### “Chimney” commando procedure

The total cardiopulmonary bypass and aortic cross-clamp times were 246.07 ± 69.83 min and 125 ± 34.66 min, respectively. Additional procedures included coronary bypass grafting in 10% (*n* = 3) of the cohort, tricuspid valve repair in 33.3% (*n* = 10), the Cox-Maze IV procedure in 16.7% (*n* = 5), and temporary pacemaker lead implantation in 100% (*n* = 30) of the cohort. Left atrial thrombectomy and left atrial appendage closure were performed in 3.3% (*n* = 1) of the patients. There were no intraoperative deaths (Table 3).

**Table 3:** Operative data.

Variables	n (%) or mean ± SD
Total CPB (min)	246.07 ± 69.83
Total aortic cross clamp (min)	155.33 ± 46.66
Combined cardiac procedure	
CABG	3 (10)
Tricuspid valve repair	10 (33.3)
Cox Maze IV	5 (16.7)
Left atrial thrombectomy	1 (3.3)
Left atrial appendage closure	1 (3.3)

CABG: coronary artery bypass grafting; CPB: cardiopulmonary bypass.

### Early outcomes

Postoperatively, the incidence of renal dysfunction requiring dialysis was 17% (*n* = 5). Other events and respective incidences were tracheostomy: 7% (*n* = 2), prolonged ventilation (>24 h): 17% (*n* = 5), atrial fibrillation: 50% (*n* = 15), reoperations due to bleeding: 3% (*n* = 1), permanent pacemaker insertion: 33% (*n* = 12), and temporary neurological impairment: 3% (*n* = 1). There were no perioperative myocardial ischaemic events. There was one hospital death (3%), due to malignant arrhythmias and multiorgan failure. The total intensive care unit (ICU) and hospital stay lengths were 7 ± 3 and 23 ± 9 days, respectively. Postoperative echocardiograms showed that the size of the aortic valve prosthesis was 24.23 ± 1.60 mm, while that of the mitral valve prosthesis was 28.33 ± 1.21 mm. Postoperative echocardiograms also showed that the PG of the aortic valve prosthesis was 18.17 ± 6.44 mmHg and that of the mitral valve prosthesis was 12.73 ± 5.45 mmHg. In all patients, steady IVF reconstruction was confirmed, with no abnormal ﬂow communication between the left heart chambers. The cohort had 83% of patients with normal left ventricle (LV) function, 10% with mild dysfunction, 3% with moderate dysfunction, and 3% with severe LV dysfunction (Table 4).

**Table 4:** Postoperative complications and early outcomes.

Variables	*n* (%) or mean ± SD
Hospital death	1 (3.3)
Reoperation for bleeding	1 (3.3)
AKI requiring dialysis	5 (16.7)
Prolonged ventilation > 24 h	5 (16.7)
Stroke	0
Tracheostomy postoperatively	2 (6.7)
Temporary neurological impairment	1 (3.3)
AF postoperatively	15 (50.0)
PPM implant	1 (3.3)
Tracheostomy postoperatively	2 (6.7)
Hospital length of stay (days)	23.44 ± 9.11
ICU length of stay (days)	7.23 ± 3.43
Echocardiography	
Aortic valve prosthesis size (mm)	24.23 ± 1.60
Mitral valve prosthesis size (mm)	28.33 ± 1.21
Aortic valve PG (mm Hg)	18.17 ± 6.44
Aortic valve MG (mm Hg)	16.71 ± 6.59
Mitral valve PG (mm Hg)	12.73 ± 5.45
Mitral valve MG (mm Hg)	10.95 ± 4.15
LV dysfunction	
None	25 (83.3)
Mild	3 (10.0)
Moderate	1 (3.3)
Severe	1 (3.3)
Ejection fraction (%)	51.45 ± 4.19

AKI, acute kidney injury; AF, atrial ﬁbrillation; PPM, permanent pacemaker; ICU, intensive care unit; PG, peak gradients; MG, mean gradients; LV, left ventricular.

### Short-term follow-up results

During the 1-month follow-up period, there was no LV dysfunction in 40% of the patients, while 40% had mild, and 20% had moderate LV dysfunction. The mean gradients (MG) of the aortic valve and the mitral valve prostheses was 15.83 ± 6.47 mmHg and 11.54 ± 4.83 mmHg, respectively. During the 6-month follow-up period, there was no LV dysfunction in 57% of the patients, while 38% had mild, and 5% had moderate LV dysfunction. The MG of the aortic valve and the mitral valve prostheses was 16.26 ± 6.44 mmHg, and 11.24 ± 4.90 mmHg, respectively. During the 1-month and 6-month follow-up periods, there were no thrombosis or periprosthetic leakage events (Table 5).

**Table 5:** Follow-up.

Variables	*n*/*N* (%) or mean ± SD	
Patients discharged	29/30 (96.7)	
Death after discharge	0	
Patients followed up at 1-month visit	5/30 (16.7)	
Patients followed up at 6-month visit	21/30 (70.0)	
Echocardiographic data	1-month	6-month
During follow-up	follow-up	follow-up
Aortic valve MG (mm Hg)	15.83 ± 6.47	16.26 ± 6.44
Mitral valve MG (mm Hg)	11.54 ± 4.83	11.24 ± 4.90
LV dysfunction		
None	2 (40.0)	12 (57.1)
Mild	2 (40.0)	8 (38.1)
Moderate	1 (20.0)	1 (4.8)
Severe	0	0
Ejection fraction (%)	55.43 ± 5.54	54.73 ± 8.32
Thrombosis	0	0
Periprosthetic leakage	0	0

MG, mean gradients; LV, left ventricle; SD, standard deviation.

## Discussion

**Figure 5:**
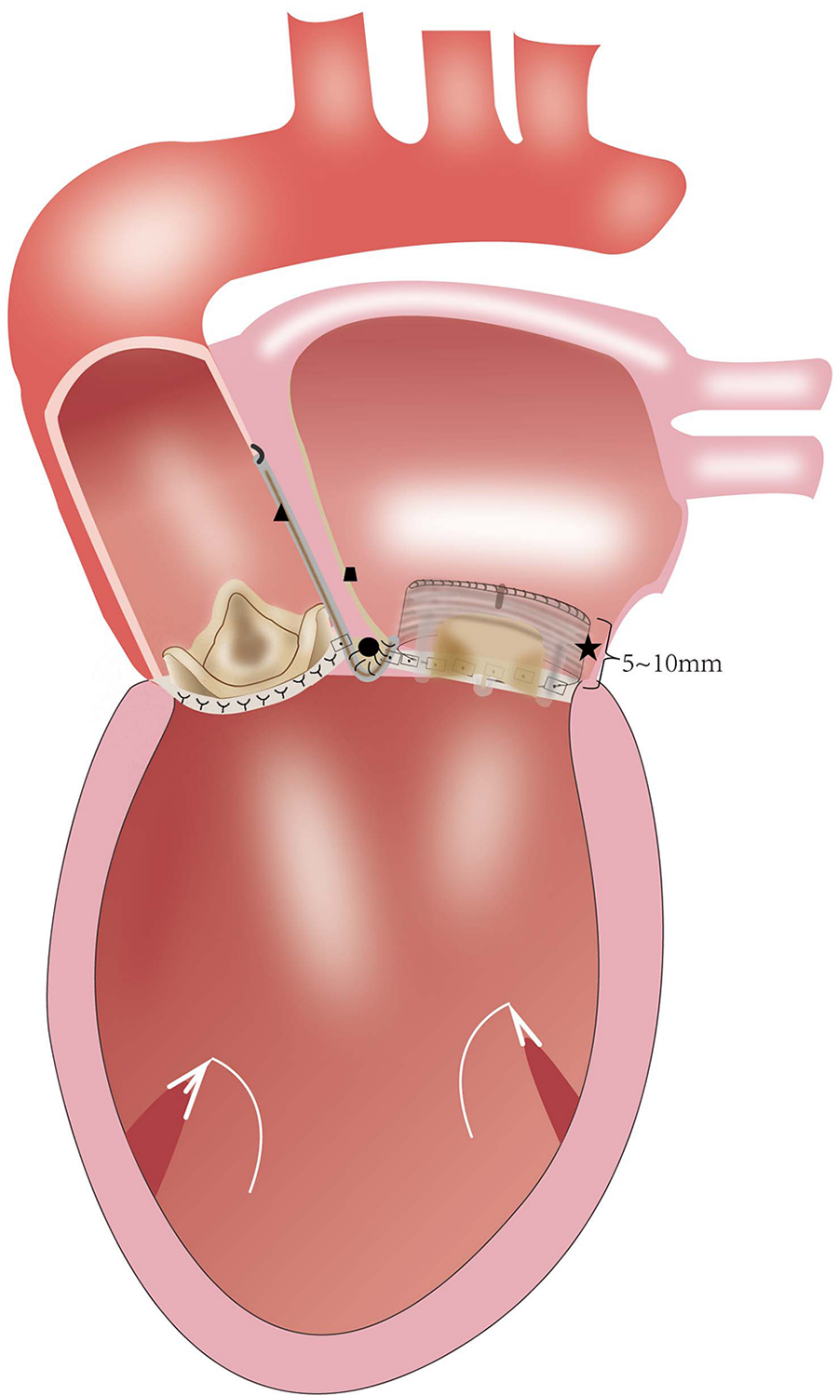
(★) valved conduit; (●) the intervalvular fibrosa of reconstruction; (▴) the woven polyester vascular patch; (∎) the patch of right atrial closure.

The Due to the extremely high complexity degree of a surgical repair, the Commando procedure is well-known for the high risk of intra-operative and post-operative complications and mortality (7–10). In patients with infective endocarditis (IE), the overwhelming majority of previous reports on this procedure have consisted of DVR combined with the reconstruction of the intervalvular fibrous body (11–16). The Commando procedure is a common choice for the surgical treatment of patients with extremely small aortic and mitral annuli. According to the Manouguian's experiment (17), if the anterior mitral continuity is enlarged by more than 30 mm (a 30 mm increase of the mitral annulus), the annulus of the mitral prosthesis protrudes into the left ventricular outflow tract (LVOT) and obstructs it. This consequence limits the size of the mitral valve prosthesis that can be inserted. Nevertheless, after IVF reconstruction and mitral valve annulus enlargement, MVR frequently fails to reconcile the ideal mitral valve size with the LVOT obstruction. The main concern is how to prevent LVOT obstruction and consequent interference with implanted valves. Tetsuro Uchida et al. (18) reported a modified Commando procedure using a stentless aortic bioprosthesis. Haytham Elgharablya et al. (11) reported the “Hemi-Commando” procedure for the treatment of double-valve IE, which extends into the anterior mitral leaflet, but spares the posterior leaflet and annulus. David et al. (19) reported the reconstruction of the left ventricular inﬂow and outﬂow tracts with 2-valved conduits, in patients with prosthetic mitral valve mismatch and/or small native mitral annulus. Our group has developed a modified surgical technique, the “Chimney” Commando procedure for the treatment of patients with small aortic root after prior valve operations. This procedure can adjust the relative position and orientation of the aortic and mitral prostheses to obtain good hemodynamics and avoid LVOT obstruction (Figure 5). By developing the “Chimney” technique, our center has innovated by upgrading the Commando procedure. Our center has adapted it to patients with small aortic and mitral valves. The previous prostheses for those patients were removed, and extensive debridement of the annulus was performed, which led to a total (or at least partial) removal of annular tissue. The skirt end of this self-assembled valvular conduit was sutured to the endocardium of the posterior wall of the left atrium, from the lateral to the medial fibrous trigones. If the patient's tissues are quite fragile, this suturing technique may lead to perivalvular leakage, or even avulsion of the valved conduit. Long-term follow-up is required to accurately assess the durability of the suturing approach. Nevertheless, periprosthetic leakage was not detected in our group after one or six months following surgery. The prevention of LVOT obstruction and implantation of an excessively small aortic valve prosthesis during the Commando procedure requires the avoidance of mitral valve prosthesis oversizing (20). This technique expands the mitral annulus to anticipated dimensions, regardless of the valve size. Moreover, it also keeps a sufficient distance between the mitral and the aortic valves without mutual interference. The proximal part of the valved conduits is trimmed and used to reconstruct the left ventricular outﬂow tract and the aortic root. Technically, the “Chimney” Commando procedure is less complex than the classic Commando procedure. The aortic cross-clamp time for most of the reported Commando procedures [ranging from 124 ± 42 min to 297 ± 71 min (8, 9, 19)] is longer than the “Chimney” Commando procedure surgery time (Table 3). Importantly, the “Chimney” Commando procedure alters the physiology of the left ventricular inflow tract, through the implanted valved conduit. This alteration inevitably affects the local hemodynamics of the left ventricular inflow and left atrial outflow tracts, which may result in thrombosis. Although thrombosis was not detected in our cohort at one and six months after surgery, a long-term follow-up is needed, in order to evaluate the hemodynamic stability of the implanted valved conduit (Table 5).

**Figure 6:**
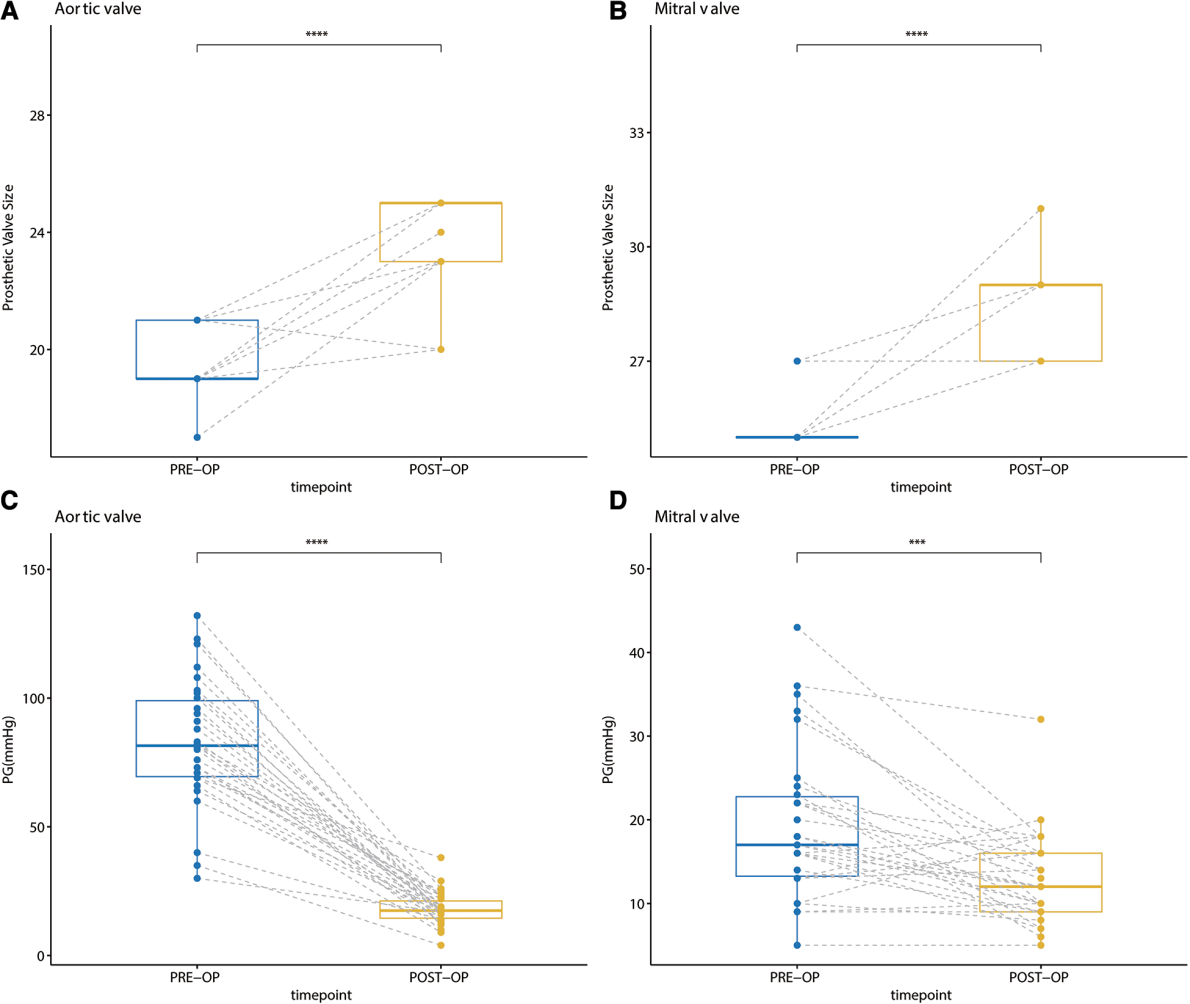
(**A**) comparison of the size of the implanted aortic valve prosthesis before and after surgery; (**B**) comparison of the size of the implanted mitral valve prosthesis before and after surgery; (**C**) comparison of the mean aortic valve gradient one month after the last DVR and “chimney” commando procedure; (**D**) comparison of the mean mitral valve gradient one month after the last DVR and “chimney” commando procedure.

The reported early mortality rates of the Commando procedure range from 6% to 32% (7, 11). In this “Chimney” Commando procedure technique, we had no operative deaths and low early mortality for the studied group of patients (Table 3). Regarding the Commando procedure postoperative complications, it has been reported that the rate of reoperations caused by bleeding was 8%, the frequency of neurological events was 3%, acute renal failure was 7%, and pacemaker insertion was 33% (19). In this “Chimney” Commando procedure series, we had lower rates of reoperation for bleeding, as well as less frequent neurological events, acute renal failure, and pacemaker implantation than in the regular Commando procedure (Table 4). Hosseini et al. (21) reported a valve prosthesis which, by enlarging both annuli, is at least twice as large as the natural annuli that can be inserted during the Commando surgery. However, the sizes of the implanted aortic and mitral valve prostheses are 24.23 ± 1.60 mm and 28.33 ± 1.21 mm, respectively. Because of the “Chimney” Commando procedure, these are significantly larger than the preoperative values (*p *<* *0.001). Notably, the patient's mean aortic and mitral valve gradients one month following the “Chimney” Commando surgery were 15.83 ± 6.47 mmHg and 11.54 ± 4.83 mmHg, respectively (Figure 6). One month after the patient's last standard DVR, these values were significantly lower than the patient's mean aortic and mitral valve gradients (*p *<* *0.001).

The limitations of this study include that this is a single-institution retrospective study of a relatively small cohort of patients with small aortic and mitral annuli undergoing surgical treatment with only early outcomes. Adjustment of the relative orientation and position of the two valve prostheses is based on the experience of the surgeon and the intraoperative situation, which requires the same surgeon to complete the procedure in this “Chimney” Commando procedure series, while with one surgeon performing the majority (85%) of operations. Additionally, this is a relatively new technique, so long-term outcomes are not available at this time.

In conclusion, the early outcomes of the “Chimney” Commando procedure suggest that it is a good alternative treatment for patients with small aortic and mitral annuli after prior valve operations. The “Chimney” Commando procedure neither limits the aortic and mitral prostheses size, nor leads to any LVOT obstruction.

## Data Availability

The raw data supporting the conclusions of this article will be made available by the authors, without undue reservation.
